# Phytochemical Screening and Evaluation of Pesticidal Efficacy in the Oleoresins of *Globba sessiliflora* Sims and *In Silico* Study

**DOI:** 10.1155/2023/5936513

**Published:** 2023-01-03

**Authors:** Bhawna Verma, Himani Karakoti, Ravendra Kumar, Sonu Kumar Mahawer, Om Prakash, Ravi Mohan Srivastava, Satya Kumar, Shilpi Rawat, Dharmendra Singh Rawat, Mozaniel Santana de Oliveira

**Affiliations:** ^1^Department of Chemistry, College of Basic Sciences and Humanities, G.B. Pant University of Agriculture and Technology, Pantnagar 263145, U.S. Nagar, Uttarakhand, India; ^2^Department of Entomology, College of Agriculture, G.B. Pant University of Agriculture and Technology, Pantnagar 263145, U.S. Nagar, Uttarakhand, India; ^3^Department of Plant Pathology, College of Agriculture, G.B. Pant University of Agriculture and Technology, Pantnagar 263145, U.S. Nagar, Uttarakhand, India; ^4^Department of Biological Sciences, College of Basic Sciences and Humanities, G.B. Pant University of Agriculture and Technology, Pantnagar 263145, U.S. Nagar, Uttarakhand, India; ^5^Campus de Pesquisa-Museu Paraense Emílio Goeldi-Botany Coordination, Av. Perimetral 1901, Terra Firme 66077830, Belem, PA, Brazil

## Abstract

*Globba sessiliflora* Sims is an aromatic rhizomatous herb of family Zingiberaceae which is endemic to Peninsular India. This study first reports the phytochemical profile and pesticidal potential of oleoresins obtained from the aerial and rhizome parts of *Globba sessiliflora* Sims. The oleoresins were prepared by the cold percolation method and were analyzed by a gas chromatography-mass spectrometry (GC-MS) method. Both the oleoresins varied greatly in composition, the major compounds identified in aerial part oleoresin (GSAO) were methyl linoleate, methyl palmitate, and phytol, while the major compounds present in rhizome part oleoresin (GSRO) were *γ*-sitosterol, 8 (17),12-labdadiene-15, 16-dial, methyl linoleate, and methyl palmitate. In order to evaluate the biological activities, the oleoresins were tested under laboratory conditions for nematicidal action and inhibition of egg hatching potential against root knot nematode, where GSRO was more effective. Insecticidal activity was performed against mustard aphid, *Lipaphis erysimi* and castor hairy caterpillar, *Selepa celtis*. In case of mustard aphid, GSRO (LC_50_ = 154.8 ppm) was more effective than GSAO (LC_50_ = 263.0 ppm), while GSAO (LC_50_ = 346.7.0 ppm) was more effective against castor hairy caterpillar than GSRO (LC_50_ = 398.1 ppm). The herbicidal activity was performed in the receptor species *Raphanus raphanistrum* subsp. *sativus*, and the oleoresins showed different intensities for seed germination inhibition and coleoptile and radical length inhibition. Molecular docking studies were conducted to screen the *in vitro* activities and through molecular docking, it was found that the major oleoresins components were able to interact with the binding pocket of HPPD and AChE with *γ*-sitosterol showing the best binding affinity.

## 1. Introduction

The need for more food to feed the World's expanding population prompted the creation and widespread use of synthetic pesticides as a rapid and efficient method of controlling agricultural pests and diseases [1]. Synthetic pesticides are the chemicals used to control and manage plant pests such as insects, weeds, bacterial, and/or fungal infections, and are commercialized in the form of herbicides, insecticides, nematicides, rodenticides, molluscicides, animal repellents, insect repellents, antimicrobials, and fungicides [2]. India ranks third in Asia and twelfth globally for pesticide use. The FAO estimates that India used about 58,160 tonnes of pesticides in 2018 which was 0.31 kg of pesticides per acre [3]. Although pesticides are useful in raising crop yields and creating affordable and high-quality food, their widespread usage has a number of adverse effects on environment and human health such as pesticide resistance, soil pollution, pesticide residues on food commodities, biomagnification, bioaccumulation, acute or chronic toxicity to humans, and other nontarget organisms [4, 5].

Secondary plant metabolites, which include phenolics, alkaloids, terpenoids, saponins, and other chemical substances, are produced by plant cells via metabolic processes. These metabolites have been identified as being involved in a wide range of biological processes, establishing the scientific framework for the use of herbs in a traditional medicine system, as antibacterial, antifungal, and antiviral agent, and hence, capable of protecting plants from pathogens. These botanical pesticides have several advantages over synthetic pesticides, including the fact that they are effective in controlling a wide range of agricultural pests, are inexpensive, easily biodegradable, have less toxicity, have a variety of mechanisms of action, are readily available in their sources, and are not harmful to nontarget organisms. The phytochemical makeup of various plants is thought to be responsible for their various ways of action. As a result, they can be utilized in integrated pest management programmes and actually produce sustainable agriculture [1].

Zingiberaceae/ginger family is one of the major families in the plant kingdom typically found in tropical and subtropical regions of the world [6]. It comprises of more than 1,400 species in about 52 genera of medicinal and aromatic perennial herbs with creeping tuberous or horizontal rhizomes. In addition to their usage as spices, cosmetics, ornamentation, food preservatives, and other products, plants in this family are utilised in traditional herbal folk medicine. Several members of this family of species include secondary metabolites that have medicinal applications and have properties such as anti-inflammatory, antimutagenic, antioxidant, antibacterial, antidiabetic, hepatoprotective, anticancer, and expectorant [7, 8].

*Globba* L. is the third largest genus in the Zingiberaceae family with over 100 species [9], typically found in tropical and subtropical Asia, from India to southern China, and south and east to the Philippines and New Guinea [10]. In India, *Globba* L. genus is represented by 17 species mainly distributed in the Himalaya, South India, and Andaman and Nicobar Islands [11]. Different *Globba* species have been used for centuries to treat a variety of diseases including postpartum, mouth ulcers, postnatal care for both mother and child, conjunctivitis, eye infections, analgesic, abrasions, asthma, leucoderma, cough, food poisoning, stomachache, fever, and heart disease [12].

The oil and the extracts of *Globba* species have been found to possess a wide range of biological activities such as anti-inflammatory, antinociceptive, antipyretic [13, 14], antioxidant [15], and antimicrobial [16]. Our laboratory has earlier reported the chemical composition of essential oil of aerial and rhizome parts of *G. sessiliflora* [12, 17]. In our continuing programme to reinvestigate the plant for their bioactive components, oleoresins were prepared from aerial parts (GSAO) and rhizomes (GSRO). Oleoresin is a viscous liquid or semisolid substance derived from finely ground powder that contains the aroma and flavour of its source [18]. The majority of the oleoresins are utilised as flavours in food and beverages, as well as in the perfume and pharmaceutical industries [19].

Molecular docking is the most suitable computational method to perform the mechanism of inhibitory action for the pharmacologically active components, which helps to understand the interactions of the enzyme with the major oil components. The virtual ligand screening also provides the information about the most appropriate geometry and binding affinity of the tested components (ligand) to the active site of the protein/enzymes.

Though the plant *Globba sessiliflora* is not widely explored for its phytochemical profiling, and there are no records in the literature related to the chemical composition and pesticidal properties of its oleoresin. As a result, the present study first reports the yield, chemical composition, and pesticidal activities of the oleoresins of aerial and root part of *G. sessiliflora*, aiming to contribute to the phytochemical knowledge of aromatic plant of the genus *Globba* from the Himalayan region. To support the *in vitro* nematicidal, insecticidal, and herbicidal activities, molecular docking studies were performed on target enzymes.

## 2. Materials and Methods

### 2.1. Collection of Plant Material

The medicinal and aromatic herb *Globba sessiliflora* was collected in the month of July, 2021 from Kausani (29.8445°N and 79.6039°E), Bageshwar, Uttarakhand, India. The plant was identified by one of the authors (D.S. Rawat), Assistant Professor and Plant Taxonomist, Department of Biological Sciences, C.B.S. & H., G.B.P.U.A. & T., Pantnagar. The specimen of *Globba sessiliflora* Sims with voucher number GBPUH-1209 was deposited at the herbarium of Department of Biological Sciences for future references.

### 2.2. Preparation of Oleoresins

The oleoresins were prepared from finely grinded shade dried aerial and rhizome parts individually by using the cold percolation method, and were designated as GSAO and GSRO, respectively. The oleoresins were prepared with successive soaking (aerial part (90 gm) and rhizomes (45 gm)) in hexane and further shaking for 48 hrs [20]. The oleoresins were filtered, and then the solvent was evaporated under vacuum on a rotatory evaporator at 45–60°C till dryness. The average percent yield (w/w) of oleoresins obtained was 3.7% and 3.3% for GSAO and GSRO, respectively. The oleoresins were stored at 4°C for further use.

### 2.3. GC-MS

The chemical constituents of oleoresins were analyzed by GC-MS (Shimadzu QP 2010 plus instrument) with GCMS-QP 2010 Ultra DB-5 and GCMS-QP 2010 Ultra Rtx-5MS columns (30 m × 0.25 mm i.d., 0.25 *µ*m). The experimental conditions are as follows: Carrier gas: helium with flow rate = 1.21 mL/min, and split ratio = 10.0. Oven temperature: 50–280°C with a temperature gradient of 3°C/min upto 210°C (isotherm for 2 min), and then 6°C/min upto 280°C. Identification of oleoresins components was performed by comparing their relative retention index (RI) values with mass spectra NIST (NIST version 2.1) and WILEY (7th edition) libraries, and also by matching the fragmentation pattern of the mass spectral data with those reported in the literature [21].

### 2.4. Pesticidal Activities

#### 2.4.1. Nematicidal and Egg Hatching Activity

*(1) In vitro Evaluation of Oleoresins Samples on Mortality of Second Stage Larvae of Meloidogyne incognita*. [22, 23]. To evaluate the impact of oleoresins on the mobility of second stage larvae of *M. incognita*, an *in vitro* experiment was conducted. The egg masses from a pure nematode culture were used to isolate the second stage larvae from galled roots. The galled roots were washed and rinsed to remove the soil adhered to its surface. It was then divided into 2 cm pieces, added to a solution of 2% sodium hypochloride, and agitated for two minutes to separate the organic matter from the eggs. A series of sieves were used to filter the suspension, and the eggs that passed through using a 38-m-pore sieve were carefully collected and cleaned with tap water. The egg solution was then added to the incubator, where it was kept at a temperature of 28°C for 48 hours to produce second-stage juveniles from the hatched eggs. Hundred juveniles in the second stage were counted and put in the gridded petri dishes with an oleoresin stock solution. Six separate doses of the solutions at 0.05%, 0.10%, 0.25%, 0.50%, 0.75%, and 1.00% were used in three duplicate treatments. Distilled water was used as a control. A random order was used to allocate each treatment. Using a stereobinocular microscope, the number of dead juveniles was counted at intervals of 24, 48, and 72 hours. The nematodes that were almost straight in position (not moving) were assumed to be dead. The percent mortality was calculated by the following formula:

(1) $$\begin{array}{c} Percent\;Mortality=\frac{Nd}{Nt}\times 100\text{,} \end{array}$$

where Nd = Total number of dead larvae; Nt = Total number of larvae.

*(2) In vitro Evaluation of GSAO and GSRO on Egg Hatching of Meloidogyne incognita*. [23, 24]. To determine the effectiveness of the oleoresins on the hatching of *M. incognita* eggs derived from nematode pure culture, an *in vitro* experiment was conducted. Petri dishes with grids were used for the experiment. There were a total of three treatments, each of which was reproduced three times and given in a range of six doses. Distilled water was taken as the control. Two *M. incognita* egg masses were suspended in oleoresin concentrations (0.05%, 0.10%, 0.25%, 0.50%, 0.75%, and 1.00%) in gridded Petri dishes. All treatments were set up in an entirely random order and maintained in the BOD incubator at a constant temperature of 27°C. Observations on egg hatchability of eggs were made under a microscope at 40*x* magnification at 24, 48, 72, and 96 hours exposer periods by counting the number of eggs hatched.

#### 2.4.2. Insecticidal Activity

The leaf dip method was used to assess the insecticidal activity of GSAO and GSRO using the standard protocols [25, 26].

*(1) Experimental Procedure for Insecticidal Activity against Mustard Aphids, Lipaphis erysimi and Castor Hairy Caterpillar, Selepa celtis*. The experiment was assessed in petri plates which were filled with agar solution (approx 1 cm thick) to maintain proper humid condition and to keep the treated leaves fresh. The mustard and castor leaves (each of area 25 sq.cm.) were dipped in the oleoresins (GSAO and GSRO) of varying concentrations (100 ppm, 250 ppm, 500 ppm, and 750 ppm) for 1 minute. Furthermore, the leaves were air dried and transferred to petri plates for testing the toxicity of oleoresins. Counted numbers of *Lipaphis erysimi* nymphs (starved for 3–4 hrs) were then released to each petri plate containing the treated leaves of mustard with various concentrations of oleoresins. Similarly, 24 hrs-starved fourth instar larvae of *Selepa celtis* were released to each petri plate containing the treated leaves of castor. Readings were noted at the time interval of 24, 48, and 72 hrs after the release of the test insect, and then, the number of dead aphids and larvae was counted. The percent mortality was calculated by the following formula:

(2) $$\begin{array}{c} Percent\;Mortality=Nt-\frac{Nd}{Nt}\times 100\text{,} \end{array}$$

where Nt = Total number of insects; Nd = Total number of dead insects.

#### 2.4.3. Herbicidal Activity

The herbicidal activity was assessed against radish seeds (*Raphanus raphanistrum* subsp. *sativus*) for the effects on different growth parameters such as percent germination, coleoptile length, and radical length [27, 28]. The radish seeds were sterilized in 1% H_2_O_2_ solution for 3-4 hrs. To evaluate the herbicidal activity of the sample, 2 ml of various levels (250, 500, 750, and 1000 ppm) was applied to petri dishes lined with two sheets of germination paper and 10 radish seeds were placed in each dish. A solution of 1% Tween-20 in distilled water served as the negative control. In a totally randomised approach, three Petri dishes for each treatment were placed as replicates. The experiment was carried out at a room temperature range of 25–28°C. Five days of following treatment, the germination rate and root and shoot lengths were measured in 24, 48, 72, 96, and 120 hrs. 1% Tween-20 in distilled water served as the negative control, whereas pendimethalin was taken as positive control. Germination percentage was calculated by the following formula:

(3) $$\begin{array}{c} Germination\%=\frac{NT}{N}\times 100\text{,} \end{array}$$

where NT = Proportion of germination seeds in each treatment for the final measurement; *N* = Number of seeds used in the bioassay.

At the end of the 120 hrs of incubation, the length of the shoot and root was measured. The formulae used for determining the inhibition of shoot and root growth are as follows:

(4) $$\begin{array}{c} Inhibition\;of\;hypocotyl\left(shoot\;length\right)growth\left(\%Inhibition\right)=100\times \left(1-\frac{St}{Sc}\right)\text{,} \end{array}$$

where St = shoot length growth in treatment and Sc = shoot length growth in control.

(5) $$\begin{array}{c} Inhibition\;of\;radicle\left(root\;length\right)growth\left(\%Inhibition\right)=100\times \left(1-\frac{Rt}{Rc}\right)\text{,} \end{array}$$

where Rt = root length growth in treatment and Rc = root length growth in control.

#### 2.4.4. Molecular Docking Studies

The molecular docking study of the selected compounds from GSAO and GSRO was carried out on 4-hydroxyphenylpyruvate dioxygenase (HPPD) receptors and acetylcholinesterase (AChE) receptors. In our results, the tested oleoresins were found to possess good postemergence herbicidal activity against the radish seeds, for which HPPD was selected as a target enzyme as it has been reported as a molecular target for compounds with postemergence herbicidal activity [29]. The oleoresins possessed good to moderate insecticidal and nematicidal activity, for which the enzyme AChE was selected. Most of the insecticides and nematicides compounds act by inhibiting AChE [30]. The three-dimensional (3D) structures of the HPPD and AChE proteins were obtained from the RCSB ProteinData Bank with PDB ID: 6J63 and 1QON, respectively. The 3D structures of the selected proteins converted into PDB formats by deleting the water molecules, HETATOMS and adding polar hydrogens using Biovia Discovery Studio-2021 Client. The compounds from the oleoresins for docking studies were selected based on their higher percentage contents. The structures of the selected compounds were downloaded from the PUBCHEM database (https://pubchem.ncbi.nlm.nih.gov/) in their SDF (structure data file) format. The selected compounds from GSAO and GSRO were methyl linoleate, methyl palmitate, *β*-pinene, phytol, germacrene D, *γ*-sitosterol, and 8(17),12-labdadiene-15,16-dial. PyRx software was used to perform the docking process. Using the open babel tool in PyRx software, the structures of the ligands were imported and energy minimization (optimization) was performed by adding charges and optimizing the universal force field. Furthermore, the ligands were saved into AutoDock Ligand format (PDBQT). Using Vina Wizard tool, the binding affinity and the various ligand-receptor interactions responsible for the herbicidal, insecticidal, and nematicidal activities were validated. The proteins and multiple ligands to be docked were selected in the PyRx software using the Vina Wizard Control tool. The “Run Vina” control was selected to initiate the docking process. By selecting the “Analyze Vina” tool, the results were observed and exported as CSV files [31] and the 2D and 3D interactions of docking poses were visualized using Biovia Discovery Studio-2021 Client.

#### 2.4.5. Statistical Analysis

The data were expressed in terms of the mean and ±standard deviation, in triplicates. Using SPSS 12.0 program, the data were subjected to ANOVA at a 1% level of significance (*p* < 0.01) for nematicidal activity and at a 5% level of significance (*p* < 0.05) for insecticidal and herbicidal activities. At each level of significance, it was found that the studied data were significantly different. OriginPro 2021 version 9.8.0.200 was used to perform principal component analysis (PCA) on the chemical composition of the oleoresins under study.

## 3. Results and Discussion

### 3.1. GC-MS Analysis

**Figure 1:**
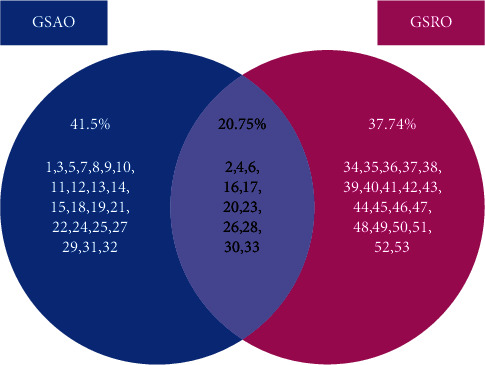
Venn diagram of oleoresins composition of GSAO and GSRO.

The comparative study of both the oleoresins together revealed that, a total of fifty-three components were found in *G. sessiliflora*. The composition of the intact plant oleoresins (GSAO and GSRO) differed significantly in both aerial part and rhizomes part (Table 1). Both the oleoresins GSAO and GSRO share a total of 11 components common in their composition as shown in the Venn diagram (Figure 1**)**.

Major compounds present in GSAO were methyl linoleate (18.2%), methyl palmitate (17.1%), *β*-pinene (10.5%), phytol (10.2%), and germacrene D (5.8%). On the other hand, the major compounds present in GSRO were *γ*-sitosterol (22.1%), 8(17),12-labdadiene-15,16-dial (18.6%), methyl linoleate (4.9%), and methyl palmitate (4.4%). The compounds which were only present in aerial part were phytol (10.2%), *β*-elemene-2 (4.9%), 1,8- linalool (3.0%), 1-nonadecene (2.4%), cineole (2.3%), phytone (1.9%), pinocarvone (1.3%), aromadendrene (1.0%), methyl-*cis*-octadec-11-enoate (1.7%), *α*-pinene (0.9%), *β*-selinene (0.6%), bicyclogermacrene (0.7%), *β*-bourbonene (0.7%), myrtenal (0.5%), *α*-ylangene (0.4%), *α*-amorphene (0.4%), 1-eicosene (0.4%), sabinene (0.3%), n-pentadecane (0.3%), terpinen-4-ol (0.3%), myrtenol (0.3%), limonene (0.2%), and isobornyl acetate (0.2%), whereas ambrial (3.3%), muurolol (3.1%), heptacos-1-ene (2.6%) *α*-8,11,14-docosatrienoic acid, methyl ester (2.3%), *epi*-*α*-muurolol (1.7%), caryophyllene oxide (0.8%), oleic acid, methyl ester (0.7%), *trans*-*p*-menthane (0.3%), bornyl acetate (0.3%), and *α*-cadinene (0.2%) were only present in the rhizome part. The chemical profile of GSAO was dominated by fatty acids (39.2%), sesquiterpenoid hydrocarbon (17.6%), and oxygenated diterpenoid (12.1%), while GSRO was dominated by steroids (25.5%), oxygenated diterpenoid (18.7%), and hydrocarbon (12.0%) (Table 1).

Compounds such as caryophyllene oxide, linalool, 8(17),12-labdadiene-15,16-dial which were present in GSAO and GSRO were also reported in other species of *Globba* as *G. schomburgkii, G. marantina*, and *G. sherwoodiana* [32–34]. The EO of *G. sessiliflora* has also been examined in earlier investigations. For instance, the compounds present in GSAO and GSRO such as *α*-pinene, sabinene, linalool, *α*-humulene, germacrene D, *β-*selinene, *δ*-cadinene, and caryophyllene oxide were also found in the essential oil of leaf and rhizome part of the plant collected from the Kumaon region of Uttarakhand [17]. Another study of EO collected from the Garhwal region of Uttarakhand revealed the presence of compounds such as sabinene, *β*-pinene, limonene, 1,8-cineole, linalool, *α*-humulene, bornyl acetate, and caryophyllene oxide, which are also found in the composition of oleoresins under investigation [35]. The chemical composition from GSAO and GSRO, according to the literature search and to the best of our knowledge, is a new chemovariant of the genus *Globba* because of its abundance in fatty acids, steroids, oxygenated diterpenoid, and oxygenated sesquiterpenoids (Table 1).

#### 3.1.1. Principle Component Analysis

**Figure 2:**
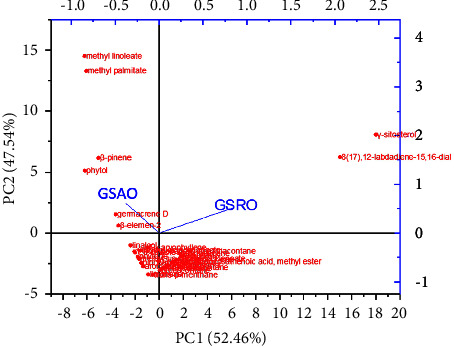
PCA of GSAO and GSRO based on their oleoresin components.

Principal component analysis (PCA), one of the best multivariate statistical methods, is used to identify the data's most important features. To assess the phytochemical heterogeneity caused by different sites' altitudes, environments, and climatic circumstances, PCA pattern recognition of oleoresins from various places was used. Chemical compositional differences, which can explain the majority of the variance information, had the only two principal components (PC1 and PC2) with a collective contribution rate of variance of 100% (Figure 2). Thus, the total compositional variability in the oleoresins was characterised by these two PCs. PC1 contributed 52.46% in the total variance, which was positively correlated with n-nonatriacontane, 1-octadecene, *α*-muurolol, ambrial, heptacos-1-ene, 8,11,14-docosatrienoic acid, methyl ester, stigmasterol, 1-tetracosanol, n-tetratriacontane, and untriacontane, whereas the contribution of PC2 to the variance is 47.54% which was positively correlated with *γ*-sitosterol and 8(17),12-labdadiene-15,16-dial.

The nature (solid/semisolid or liquid), polarity, the type of soil in which the plant grows, abiotic factors (temperature, sunlight, and pressure), and biotic factors (microflora and fauna), harvesting/collection time, extraction time/method, and various ecological niche of collection sites, may all contribute to changes in the chemical profile of hydrodistilled essential oils and oleoresins [36]. The major compounds identified in both GSAO and GSRO such as methyl linoleate, methyl palmitate, *β*-pinene, phytol, *γ*-sitosterol, and 8(17), 12-labdadiene-15,16-dial have been reported to possess various biological activities such as insect attractant, antioxidant, anti-inflammatory, anticoagulant, antiapoptotic, antifibrotic, vasodilatation properties, antibiotic resistance modulation, antitumor, antimalarial, antimicrobial, metabolism-modulating, cytotoxic, antioxidant, autophagy, anticancer, apoptosis-inducing, antihyperlipidemic, antidiabetic, antipyretic, and antitumor [37–45].

### 3.2. Nematicidal Activity of GSAO and GSRO

GSAO and GSRO showed good nematicidal activity and suppression of egg hatching in a dose-dependent manner. With an increase in oleoresin concentration, it was found that the % mortality of the nematodes increased. The mortality also increased with time reaching maximum at 72 hrs. For GSAO, minimum mortality was observed at 0.05% concentration which was increased to 11.0%, 17.3%, and 21.3% after 24, 48, and 72 hrs, respectively. The mortality increased with increase in concentration and was observed to be highest at 1.0% showing 45.3%, 52.0%, and 95.0% at the end of 24, 48, and 72 hrs, as shown in Table 2. For GSRO, the mortality was also found to be concentration dependent being minimum at 0.05% (14.0%, 42.0%, and 50.3% after 24, 48, and 72 hrs) and maximum at the highest concentration 1.0% (51.3%, 74.6%, and 96.6% after 24, 48, and 72 hrs of exposure, respectively) as shown in Table 2.

Both oleoresins produced dose- and time-dependent responses when the suppression of egg hatching was evaluated. The mean egg hatching rate for GSAO was reported to be 62.58 at the lowest concentration of 0.05% and continued to decline as the concentration was raised to 0.75% and 1%, with egg hatching rates of 10.75 and 2.5, respectively, as indicated in Table 3. The data show that egg hatching inhibition increased when concentration was raised. For the GSRO also, maximum egg hatching was observed at 0.05% concentration with a mean value of 49.08 showing maximum egg hatching inhibition. At higher concentrations, lower egg hatching percentage was observed with a mean value of 1.08 at 1.0% concentration demonstrating a higher inhibition of egg hatching (Table 4).

In the previous studies, compounds such as 1,8-cineole, limonene, methyl palmitate, selin-6-en-4-ol, germacrene B, germacrene D, *γ*-elemene, methyl linoleate, phytol, *γ*-sitosterol, *α*-pinene, and *β*-pinene present in GSAO and GSRO have shown good to moderate nematicidal activity against *Meloidogyne spp.*, *B. xylophilus* (pine-wood nematode), *Caenorhabditis elegans*, *Haemonchus contortus, Heterodera zeae*, and *Nacobbus aberrans* [43–49].

The first stage (L_1_) larvae of a nematode's life cycle result from embryonation inside an egg. The larva grows and moults through three larval stages (L_2_–L_4_), enlarging and losing its cuticle at each step until it becomes an adult capable of sexual reproduction. Inside the egg, Meloidogyne spp. goes through their first moult. In addition, the lipid, chitin, vitelline layers (which are primarily formed of glycoproteins), and uterine layers that make up the nematode eggshell's multilayered, robust barrier create a selective permeability that allows the applied chemical to pass through the egg [50]. Several hypotheses have been advanced to explain the nematicidal effects of plant extracts, including breakdown of cell membrane permeability and disruption of its functions, irreversible alterations of protein structures from the nematode surface caused by aldehydes, inhibition of acetylcholinesterase with neurotransmitter build-up in the nematode's central nervous system, followed by convulsion, paralysis, and death [51, 52]. On the basis of the results obtained in the present study, we can say that the reduction of the egg hatching process and high mortality of *J*_2_ of *M. incognita* may be associated with the chemical composition of the oleoresins (GSAO and GSRO).

### 3.3. Insecticidal Activity

#### 3.3.1. Insecticidal Activity of GSAO and GSRO on Mustard aphid, *Lipaphis erysimi*

GSAO and GSRO were made to apply on the mustard leaf using different concentrations (100–750 ppm) for the period of 24, 48, and 72 hrs. The percent mortality of the 2^nd^ instar nymphs of mustard aphid was found to increase with increase in concentration of the oleoresins. The percent mortality also increased with time reaching maximum at 72 hrs. For GSAO, minimum percent mortality was observed at 100 ppm concentration which was 16.6%, 33.3%, and 46.6% after 24, 48, and 72 hrs, respectively. The percent mortality increased with increase in concentration and was observed to be highest at 750 ppm showing 45.3%, 52.0%, and 95.0% at the end of 24, 48, and 72 hrs (Table 5). For GSRO, the mortality was also found to be concentration dependent being minimum at 100 ppm (26.6%, 43.3%, and 70.0% after 24, 48, and 72 hrs) and maximum at the highest concentration 750 ppm (46.6%, 70.0%, and 96.6% after 24, 48, and 72 hrs of exposure, respectively) (Table 6).

#### 3.3.2. Insecticidal Activity of GSAO and GSRO on Castor Hairy Caterpillar (*Selepa celtis*)

GSAO and GSRO were made to apply on the castor leaf using different concentrations (100–750 ppm) for the period of 24, 48, and 72 hrs. The percent mortality of the 2^nd^ stage larvae of castor hairy caterpillar was found to increase with increase in concentration of the oleoresins. The mortality also increased with time reaching maximum at 72 hrs. For GSAO, minimum mortality was observed at 100 ppm concentration which was 13.3%, 26.6%, and 46.6% after 24, 48, and 72 hrs, respectively. The mortality increased with increase in concentration and was observed to be highest at 750 ppm showing 46.6%, 66.6%, and 86.6% at the end of 24, 48, and 72 hrs (Table 7). For GSRO, the mortality was also found to be concentration dependent being minimum at 100 ppm (13.3%, 26.6%, and 46.6% after 24, 48, and 72 hrs) and maximum at the highest concentration 750 ppm (46.6%, 73.3%, and 93.3% after 24, 48, and 72 hrs of exposure, respectively) (Table 8).

Plant-based insecticides may act by producing an impermeable film on the cuticle of the insect that causes asphyxia which further causes poisoning in insects through inhalation or direct contact. The function of cellular membranes and oxidative phosphorylation can be impacted by some volatile substances that can pass through the cuticle. Furthermore, volatile substances have been shown to block the GABA (gamma-aminobutyric acid) receptor in insects. Since, oleoresins are complexed mixtures of number of compounds; their whole biological activity is hard to be explained. The major and minor compounds can interact synergistically or antagonistically to create an additive and effective system to contribute to the activity. The compounds such as methyl linoleate, methyl palmitate, *β*-pinene, *γ*-sitosterol, 8(17),12-labdadiene-15,16-dial, 1,8-cineole, and caryophyllene can be responsible for the good insecticidal activity as these have been reported to possess fumigant, repellent, and insecticidal activities [33, 53–60].

### 3.4. Herbicidal Activity

#### 3.4.1. Herbicidal Assay of GSAO and GSRO on Raddish, *R. sativus*

The inhibition of seed germination was assessed as the measure of herbicidal activity. The number of seeds germinated was counted at various concentration range of 250, 500, 750, and 1000 ppm. The average number of seeds germinated was found to be decreasing with increasing in concentration of the oleoresins. For GSAO, the average number of seeds germinated was 1.6, 1.0, 0.6, and 0 on the day 1 with the increasing concentration from 250 ppm to 1000 ppm. The seed germination decreased with increasing concentration being 5.6, 3.3, 2.6, and 0.3 on the last day (120 hrs) of experiment. The percent inhibition was found to be 64.2% at lowest concentration of 250 ppm and 97.8% at 1000 ppm (Table 9). For GSRO, the average number of seed germinated was 3.3, 1.3, 0.6, and 0.3 on the day 1 with the increasing concentration from 250 ppm to 1000 ppm. The seed germination decreased with increasing concentration being 5.6, 4.0, 2.0, and 1.0 on the last day (120 hrs) of experiment. The percent inhibition was found to be 51.8% at lowest concentration of 250 ppm and 94.8% at 1000 ppm (Table 10).

Pendimethalin, a commercial herbicide (Positive control), and distilled water (Negative control) were used for the comparison of results. The percent inhibition for the pendimethalin was found to be 92.6% at lowest concentration (250 ppm) and 97.3% at the highest concentration (1000 ppm) which is nearly same as the percent inhibition of GSAO at the same concentration.

The shoot and root length of radish was noted at the end of the experiment. The growth was inversely proportional to the concentration. For GSAO, the mean shoot and root length was 7.4 cm and 2.5 cm at 250 ppm concentration which was only 0.3 cm (root and shoot length) at the highest concentration of 1000 ppm. For GSRO, the mean shoot and root length was 7.9 cm and 3.0 cm at 250 ppm concentration which was only 0.6 cm (root and shoot length) at the highest concentration of 1000 ppm. The mean root and shoot length was noted to be 11.2 cm and 18.3 cm.

These plant-based herbicides generally inhibit plant phosphorylation by inhibiting glutamine synthase, resulting in an increase in ammonia [61]. Multiple modes of action contribute to the phytotoxic effects of plant-based pesticides, including suppression of cell division, reduction of mitochondrial respiration, reduction of photosynthetic pigments and photosynthesis, excessive production of radical oxygen species and oxidative impairment, breakdown of the waxy cuticular layer, suppression of the action of the enzymes, water uptake, and modification of the gibberellic acid concentration. The inhibition effect of oleoresins could be due to the presence of phytotoxic compounds such as *β*-caryophyllene, 1,8-cineole, methyl linoleate, methyl palmitate, *β*-pinene, phytol, *γ*-sitosterol, 8 (17), 12-labdadiene-15,16-dial, *α*-pinene, limonene, linalool, myrtenal, terpen-4-ol, and bornyl acetate that are the main components in essential oils possessing phytotoxic activity [62–65].

### 3.5. Molecular Docking Studies

**Figure 3:**
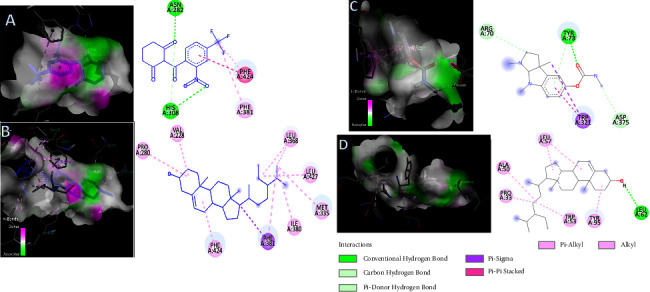
Docked conformations of molecules in the binding cavity of HPPD (PDB: 6J63) and acetylcholinesterase (PDB: 1QON) with maximum binding scores; the complex established are (a) HPPD-NTBC, (b) HPPD-*γ*-sitosterol, (c) AChE-physostigmine, and (d) AChE-*γ*-sitosterol.

The binding mode of the selected compounds with the crystal structures of the target proteins was depicted using molecular docking studies. Among all the selected compounds, *γ*-sitosterol showed the best binding affinity for HPPD and AChE with docking score −10.4 kcal/mol and −9.0 kcal/mol, respectively, which also supports the results observed in *in vitro* studies. *γ*-Sitosterol can be considered to be responsible for the greater effect of root oleoresin than the aerial part. As depicted in Table 11, all the selected compounds achieved favourable accepted scores with the two targeted proteins. For comparison purpose, the two proteins were also docked with their known inhibitors. Nitisinone (2-[2-nitro-4-(trifluoromethyl)benzoyl]cyclohexane-1,3-dione, (NTBC)) is a known inhibitor of HPPD and the binding energy for NTBC complexed with HPPD came out to be −8.9 kcal/mol, which was more than that of *γ*-sitosterol (−10.4 kcal/mol). The lower value of binding free energy indicates high docking score, representing more significant interaction between the target protein and the ligand. On the other hand, AChE was docked with its known inhibitor, physostigmine with binding energy −7.4 kcal/mol. Other compounds such as germacrene D, *γ*-sitosterol, and 8(17),12-labdadiene-15,16-dial also showed significant interaction with the amino acid residues of AChE with good docking scores. The listed binding energies of the formed complex between the selected compounds with HPPD and AChE were found to be in the range of 10.4−5.2 kcal/mol and 9.0−5.6 kcal/mol, respectively (Table 11). The more the negative values of binding free energy, more significant will be the interaction between the receptor and ligand, and hence, more will be the docking score. Based on the observation, it was found that the selected compounds interacted favorably with the receptors which reveal that the compounds can be good phytotoxic, insecticidal, and nematicidal agents. Figures 3(a)–3(d) represent the 2D and 3D interactions of selected volatiles with the amino acid residues of target proteins with highest docking score (i.e., with least binding energies).

## 4. Conclusion

*G. sessiliflora* is one such species in the genus *Globba* that has not been widely studied, especially in the form of oleoresins and its potential as a botanical pesticide. Current research was made to examine and evaluate the pesticidal efficacy of the herb *G. sessiliflora*, introducing a potential plant that can be used to develop effective formulations as a pest control agent. Moreover, in the current study, the oleoresin from the root part was found to be more effective than the aerial part which justifies its recommendation for use as pest control agent. By the analysis of ligand recognition, we have proved by molecular docking that the major compounds present in the oleoresins can be good insecticidal, nematicidal, and herbicidal agents. Altogether, this study unveiled some interesting biological activities of these oleoresins, which justify their use as botanical pesticide and open a new field of investigation for characterizing molecules involved in these processes.

## Figures and Tables

**Table 1:** Chemical diversity between GSAO and GSRO.

S. no.	Compound names	KI	*% Contribution*	
			GSAO	GSRO
1	*α*-Pinene (MH)	939	0.9	—
2	Camphene (MH)	954	0.1	0.5
3	Sabinene (MH)	975	0.3	—
4	*β*-Pinene (MH)	979	10.5	1.5
5	Limonene (MH)	1029	0.2	—
6	1,8-Cineole (OM)	1013	2.3	1.7
7	Linalool (OM)	1096	3.0	—
8	Pinocarvone (OM)	1164	1.3	—
9	Terpinen-4-ol (OM)	1177	0.3	—
10	Myrtenal (OM)	1195	0.5	—
11	Myrtenol (OM)	1194	0.3	—
12	Isobornyl acetate (OM)	1286	0.2	—
13	*α*-Ylangene (SH)	1375	0.4	—
14	*β*-Bourbonene (SH)	1388	0.7	—
15	*β*-Elemen-2 (SH)	1390	4.9	—
16	E-caryophyllene (SH)	1419	2.1	1.1
17	*α*-Humulene (SH)	1454	0.7	1.3
18	Aromadendrene (SH)	1441	1.0	—
19	*α*-Amorphene (SH)	1484	0.4	—
20	Germacrene D (SH)	1481	5.8	0.3
21	*β*-Selinene (SH)	1490	0.6	—
22	Bicyclogermacrene (SH)	1500	0.7	—
23	*δ*-Cadinene (SH)	1523	0.3	0.5
24	1-Eicosene (H)	1988	0.4	—
25	*n*-Pentadecane (H)	1500	0.3	—
26	1-Octadecene (H)	1790	0.3	3.1
27	Phytone (OD)	1790	1.9	—
28	Methyl palmitate (FA)	1921	17.1	4.4
29	1-Nonadecene (H)	—	2.4	—
30	Methyl linoleate (FA)	2085	18.2	4.9
31	Methyl cis-octadec-11-enoate (FA)		1.7	—
32	Phytol (OD)	1943	10.2	—
33	Methyl octadecanoate (FA)	2125	2.2	0.5
34	Trans-p-menthane (MH)	979	—	0.3
35	Bornyl acetate (FA)	1285	—	0.3
36	*α*-Cadinene (SH)	1538	—	0.2
37	Caryophyllene oxide (OS)	1583	—	0.8
38	Epi-*α*-muurolol (OS)	1642	—	1.7
39	*α*-Muurolol (OS)	1646	—	3.1
40	Ambrial (OS)	1800	—	3.3
41	8,11,14-docosatrienoic acid, methyl ester (FA)	2499	—	2.3
42	Oleic acid, methyl ester (FA)	1680	—	0.7
43	Heptacos-1-ene (H)	—	—	2.6
44	8(17),12-Labdadiene-15,16-dial (OD)	—	—	18.6
45	1-Tetracosanol (FA)	—	—	1.8
46	Phytol acetate (OD)	2218	—	0.1
47	Untriacontane (H)	3100	—	1.0
48	n-Nonatriacontane (H)	—	—	3.9
49	n-Tetratriacontane (H)	3400	—	1.4
50	Stigmasterol (steroid)	—	—	2.2
51	*γ*-Sitosterol (steroid)	3194	—	22.1
52	Globulol (OS)	1590	—	0.4
53	Cholestenone (steroid)	—	—	1.2
	Total		92.2	87.0

KI-Kovats indices, M+ = molecular ion peak, GSAO-*G. sessiliflora* aerial part oleoresin, GSRO-*G. sessiliflora* rhizome oleoresin, MH = monoterpenoid hydrocarbon, OS = oxygenated sesquiterpenoid, SH = sesquiterpenoid hydrocarbon, OD = oxygenated diterpene, FA = fatty acid H = hydrocarbon.

**Table 2:** Effect of GSAO and GSRO on mortality of second stage juveniles of *M. incognita*.

GSAO concentrations (%)	*Percent mortality (%)*			Mean mortality (%)	GSRO concentrations (%)	*Percent mortality (%)*			Mean mortality (%)
	24 hrs	48 hrs	72 hrs			24 hrs	48 hrs	72 hrs	
0.05	11.0 ± 1.0	17.3 ± 0.5	21.3 ± 1.5	16.2	0.05	14.0 ± 2.0	42.0 ± 1.7	50.3 ± 0.5	35.4
0.10	30.0 ± 1.0	51.6 ± 1.5	55.6 ± 1.1	45.7	0.10	17.0 ± 1.0	43.3 ± 1.5	61.3 ± 1.5	40.5
0.25	36.0 ± 1.0	45.6 ± 1.1	47.6 ± 0.5	43.1	0.25	23.3 ± 1.5	50.0 ± 1.0	72.6 ± 1.1	48.6
0.50	45.3 ± 1.5	46.3 ± 2.0	57.3 ± 1.5	49.6	0.50	43.0 ± 1.7	65.3 ± 0.5	76.0 ± 1.0	61.4
0.75	40.6 ± 1.1	44.6 ± 1.5	62.0 ± 1.7	49.1	0.75	45.0 ± 1.0	66.3 ± 1.5	77.3 ± 2.0	62.8
1.00	45.3 ± 1.5	52.0 ± 1.0	95.0 ± 3.0	64.1	1.00	51.3 ± 1.5	74.6 ± 0.5	96.6 ± 1.5	70.8
Control	1.0 ± 1.0	1.33 ± 0.5	2.3 ± 0.5		Control	1.0 ± 1.0	1.33 ± 0.5	2.33 ± 0.5	
CD 1%	1.293400	1.975701	3.422015		CD 1%	1.095279	1.673066	2.897836	
CD 5%	0.9673904	1.477137	2.559475		CD 5%	0.8192071	1.251359	2.167418	
C.V.	4.028695				C.V.	2.864411			

Results obtained using two factor CRD analysis (*p* < 0.05), GSAO = *G. sessiliflora* Aerial part oleoresin, GSRO = *G. sessiliflora* rhizome oleoresin, CD = critical difference, and C.V. = coefficient of variance.

**Table 3:** Effect of GSAO on egg hatching of *Meloidogyne incognita*.

GSAO concentrations (%)	*Average number of eggs hatched in time*				Mean eggs hatched
	24 hrs	48 hrs	72 hrs	96 hrs	
0.05	39.0 ± 4.3	54.3 ± 3.0	67.3 ± 4.7	89.6 ± 3.0	62.5
0.10	32.3 ± 5.5	41.3 ± 4.5	48.0 ± 5.0	60.3 ± 4.0	45.5
0.25	20.3 ± 2.0	31.3 ± 3.0	37.0 ± 4.0	49.3 ± 6.1	34.5
0.50	15.6 ± 2.5	24.3 ± 2.5	34.3 ± 3.2	44.3 ± 3.5	29.6
0.75	5.3 ± 1.5	7.6 ± 1.5	13.0 ± 4.0	17.0 ± 4.5	10.7
1.00	1.3 ± 1.5	2.3 ± 1.5	3.0 ± 1.0	3.3 ± 0.5	2.5
Control	90.6 ± 6.4	128.6 ± 3.5	153 ± 4.5	173 ± 4.5	
CD 1%	3.101394	4.102758	8.205517		
CD 5%	2.329842	3.082092	6.164183		
C.V.	3.196862				

Results obtained using two factor CRD analysis (*p* < 0.05), GSAO = *G. sessiliflora* Aerial part oleoresin, CD = critical difference, and C.V. = coefficient of variance.

**Table 4:** Effect of GSRO on egg hatching of *Meloidogyne incognita.*

Concentrations (%)	*Average number of eggs hatched in time*				Mean eggs hatched
	24 hrs	48 hrs	72 hrs	96 hrs	
0.05	28.0 ± 4.0	41.3 ± 3.0	56.0 ± 3.6	71.0 ± 3.6	49.08
0.10	16.0 ± 2.6	24.6 ± 23.2	37.3 ± 5.0	54.6 ± 2.5	33.16
0.25	11.3 ± 3.0	18.0 ± 2.0	28.0 ± 4.0	44.6 ± 3.2	25.5
0.50	6.6 ± 3.0	14.6 ± 3.0	23.3 ± 4.1	32.0 ± 2.0	19.1
0.75	3.3 ± 1.5	7.0 ± 1.5	12.0 ± 2.0	16.3 ± 1.5	9.6
1.00	0.3 ± 0.5	1.0 ± 0.5	1.3 ± 0.5	1.6 ± 0.5	1.08
Control	90.6 ± 6.4	128.6 ± 3.5	153.0 ± 4.5	173.0 ± 4.5	136.3
CD 1%	2.631663	3.481363	6.962726		
CD 5%	1.976969	2.615284	5.230568		
C.V.	3.169611				

Results obtained using two factor CRD analysis (*p*  < 0.05), GSRO = *G. sessiliflora* rhizome oleoresin, CD = critical difference, and C.V. = coefficient of variance.

**Table 5:** Insecticidal activity of GSAO on mustard aphid (*Lipaphis erysimi*).

Oleoresins	Concentrations (in ppm)	No. of insects used	*Average number of insects dead in time*			*Average mortality %*			Mean mortality (%)
			24 hrs	48 hrs	72 hrs	24 hrs	48 hrs	72 hrs	
GSAO	100	10	1.6 ± 0.5	3.3 ± 1.1	4.6 ± 1.5	16.6	33.3	46.6	32.2
	250	10	2.0 ± 1.0	3.6 ± 0.5	5.3 ± 1.1	20.0	36.6	53.3	36.6
	500	10	3.3 ± 0.5	5.3 ± 1.5	7.3 ± 1.5	33.3	53.3	73.3	53.3
	750	10	4.0 ± 1.0	7.3 ± 0.5	9.3 ± 1.1	40.0	73.3	93.3	68.8
	Control	10	0	0.3 ± 0.5	1.0 ± 0	0	3.3	10	6.65
CD 1%			0.9929639	1.281911	2.220335				
CD 5%			0.7374050	0.951985	1.648888				
C.V.			5.28249						

**Table 6:** Insecticidal activity of GSRO on mustard aphid (*Lipaphis erysimi*).

Oleoresins	Concentrations (in ppm)	No. of insects used	*Average number of insects dead in time*			*Average mortality %*			Mean mortality (%)
			24 hrs	48 hrs	72 hrs	24 hrs	48 hrs	72 hrs	
GSRO	100	10	2.6 ± 0.5	4.3 ± 0.5	7.0 ± 1.0	26.6	43.3	70.0	46.6
	250	10	3.0 ± 1.0	5.0 ± 1.0	8.3 ± 0.5	30.0	50.0	83.3	54.4
	500	10	3.3 ± 1.5	5.6 ± 1.1	9.0 ± 1.0	33.3	56.6	90.0	60.0
	750	10	4.6 ± 0.5	7.0 ± 1.0	9.6 ± 0.5	46.6	70.0	96.6	71.1
	Control	10	0	0.3 ± 0.5	1.0 ± 0	0	3.3	10	6.65
CD 1%			0.8468025	1.093217	1.893508				
CD 5%			0.6288611	0.811856	1.406176				
C.V.			3.81565						

Results obtained using two factor CRD analysis (*p*  < 0.05), GSAO = *G. sessiliflora* aerial part oleoresin, GSRO = *G. sessiliflora* rhizome oleoresin, CD = critical difference, and C.V. = coefficient of variance.

**Table 7:** Insecticidal activity of GSAO on castor hairy caterpillar (*Selepa celtis*).

Oleoresins	Concentrations (in ppm)	No. of insects used	*Average number of insects dead in time*			*Average mortality %*			Mean mortality (%)
			24 hrs	48 hrs	72 hrs	24 hrs	48 hrs	72 hrs	
GSAO	100	5	0.6 ± 0.5	1.3 ± 0.5	2.3 ± 0.5	13.3	26.6	46.6	28.8
	250	5	1.3 ± 0.5	2.3 ± 0.5	3.3 ± 0.5	26.6	46.6	66.6	46.6
	500	5	2.0 ± 0	2.6 ± 0.5	3.3 ± 0.5	40.0	53.3	66.6	53.3
	750	5	2.3 ± 0.5	3.3 ± 0.5	4.3 ± 0.5	46.6	66.6	86.6	66.6
	Control	5	0	0.3 ± 0.5	0.6 ± 0.5	0	6.6	13.3	9.95
CD 1%			0.53973	0.69679	1.20687				
CD 5%			0.40082	0.51745	0.896265				
C.V.			6.5788						

**Table 8:** Insecticidal activity of GSRO on castor hairy caterpillar (*Selepa celtis*).

Oleoresins	Concentrations (in ppm)	No. of insects used	*Average number of insects dead in time*			*Average mortality %*			Mean mortality (%)
			24 hrs	48 hrs	72 hrs	24 hrs	48 hrs	72 hrs	
GSRO	100	5	0.6 ± 0.5	1.3 ± 0.5	2.3 ± 0.5	13.3	26.6	46.6	28.8
	250	5	1.0 ± 0	1.6 ± 0.5	2.6 ± 0.5	20.0	33.3	53.3	35.5
	500	5	1.6 ± 0.5	2.6 ± 0.5	3.6 ± 0.5	33.3	53.3	73.3	53.3
	750	5	2.3 ± 0.5	3.6 ± 0.5	4.6 ± 0.5	46.6	73.3	93.3	71.1
	Control	5	0	0.3 ± 0.5	1.0 ± 0.5	0	6.6	13.3	9.95
CD 1%			0.53973	0.69679	1.2068				
CD 5%			0.40082	0.51745	0.8962				
C.V.			5.4849						

Results obtained using two factor CRD analysis (*p*  < 0.05), GSAO = *G. sessiliflora* aerial part oleoresin, GSRO = *G. sessiliflora* rhizome oleoresin, CD = critical difference, and C.V. = coefficient of variance.

**Table 9:** Herbicidal activity of *Globba sessiliflora* oleoresins (GSAO and GSRO) against *R. raphanistrum* in the laboratory conditions.

Samples (ppm)	*Number of seed germinated*					Mean	Percent inhibition
	24 hrs	48 hrs	72 hrs	96 hrs	120 hrs		
*(1) GSAO*							
250	1.6 ± 0.5	2.3 ± 0.5	2.6 ± 0.5	4.0 ± 1.0	5.6 ± 0.5	3.2	64.2
500	1.0 ± 1.0	1.3 ± 0.5	1.3 ± 0.5	2.0 ± 0.5	3.3 ± 1.1	1.8	80.2
750	0.6 ± 0.5	1.0 ± 0	1.3 ± 0.5	1.6 ± 0.5	2.6 ± 0.5	1.4	83.9
1000	0 ± 0	0 ± 0	0.3 ± 0.5	0.3 ± 0.5	0.3 ± 0.5	0.2	97.8
Control	7.3 ± 0.5	8.3 ± 0.5	10.0 ± 0	10.0 ± 0	10.0 ± 0	9.1	0
C.D. 1%	0.59747	0.59747	1.33599				
C.D. 5%	0.44813	0.44813	1.00206				
C.V.	3.25452						

*(2) GSRO*							
250	3.3 ± 0.5	3.6 ± 0.5	4.6 ± 1.1	4.6 ± 1.1	5.6 ± 0.5	4.4	51.8
500	1.3 ± 0.5	2.0 ± 0	3.0 ± 1.0	3.3 ± 1.1	4.0 ± 1.0	2.7	70.0
750	0.6 ± 0.5	1.0 ± 0	1.3 ± 0.5	1.3 ± 0.5	2.0 ± 0	1.2	86.1
1000	0.3 ± 0.5	0 ± 0.5	0.3 ± 0.5	0.6 ± 0.5	1.0 ± 0	0.4	94.8
Control	7.3 ± 0.5	8.3 ± 0.5	10.0 ± 0	10.0 ± 0	10.0 ± 0	9.1	0
CD 1%	0.60805	0.60805	1.35964				
CD 5%	0.45606	0.45606	1.01979				
C.V.	4.33714						

*(3) Pendimethalin*							
250	0 ± 0	0.3 ± 0.1	0.6 ± 0.5	1.0 ± 0	1.6 ± 0	0.7 ± 0.5	92.6
500	0 ± 0	0.3 ± 0.1	0.6 ± 0.5	1.0 ± 0	1.3 ± 0	0.6 ± 0.5	93.3
750	0 ± 0	0 ± 0	0.3 ± 0.1	0.6 ± 0.5	1.0 ± 0.5	0.4 ± 0	96.0
1000	0 ± 0	0 ± 0	0 ± 0	0.3 ± 0.1	1.0 ± 0.5	0.2 ± 0	97.3

Results obtained using two factor CRD analysis (*p*  < 0.05), CD = critical difference, C.V. = coefficient of variance, ppm = parts per million, GSAO = *Globba sessiliflora* aerial part oleoresin, and GSRO = *Globba sessiliflora* rhizome oleoresin.

**Table 10:** Root and shoot length growth inhibition of *R. raphanistrum* seeds by GSAO and GSRO.

Concentrations. (ppm)	Mean root length (cm)	Root growth inhibition (%)	Mean shoot length (cm)	Shoot growth inhibition (%)
*(1) GSAO*				
250	2.5	77.3	7.4	59.3
500	2.0	81.5	6.3	65.2
750	1.3	87.9	3.5	80.5
1000	0.3	96.7	0.3	97.9
Control	11.2	0	18.3	0
	CD 1%	0.359419	CD 1%	0.849543
	CD 5%	0.252871	CD 5%	0.597702
	C.V.	4.187870	C.V.	4.568621

*(2) GSRO*				
250	3	73.2	7.9	56.6
500	2.3	79.1	7.1	61.2
750	1.5	86.6	4.4	75.5
1000	0.6	94.6	0.6	96.7
Control	11.2	0	18.3	0
	CD 1%	0.275230	CD 1%	0.585747
	CD 5%	0.193692	CD 5%	0.412104
	C.V.	3.007613	C.V.	2.950383

*(3) Pendimethalin*				
250	0.4	96.2	0.1	99.2
500	0.1	98.3	0.01	99.9
750	0	100	0	100
1000	0	100	0	100
	CD 1%	0.273676	CD 1%	0.768356
	CD 5%	0.198734	CD 5%	0.535864
	C.V.	2.651287	C.V.	7.554656

Results obtained using one factor CRD analysis (*p*  < 0.05), CD = critical difference, C.V. = coefficient of variance, GSAO = *Globba sessiliflora* aerial part oleoresin, and GSRO = *Globba sessiliflora* rhizome oleoresin.

**Table 11:** Binding free energy values calculated through the molecular docking of the major volatiles identified in GSAO and GSRO for HPPD and AChE receptors.

Compounds	Interacting residue (HPPD, PDB: 6J63)	Binding energy (−kcal/mol)	Compounds	Interacting residue (AChE, PDB: 1QON)	Binding energy (−kcal/mol)
Methyl linoleate	Alkyl: Met335 (4.22 Å) Leu427 (4.45 Phe424 (4.31 Å), Pi-alkyl: Phe381 (4.57 Å), Phe424 (4.31 Å), Phe392 (4.68 Å)	−5.7	Methyl linoleate	H bond: Trp472 (3.06 Å), Trp83 (2.92 Å), C-H bond: Asp482 (3.68 Å), Pi-alkyl: Tyr374 (3.90 Å), Tyr71 (3.72 Å), Tyr370 (4.84 Å), Phe371 (5.27 Å), Phe330 (5.22 Å)	−7.2
Methyl palmitate	Alkyl: Leu427 (4.73 Å), Leu368 (5.31 Å), Pi-alkyl: Phe424 (4.53 Å), Phe381 (3.99 Å), Phe392 (5.43 Å), His308 (4.91 Å), H-bond: Asn282 (2.02)	−5.2	Methyl palmitate	C-H bond: Glu69 (3.67 Å), Pi-sigma: Tyr71(4.79 Å), Tyr324 (4.89 Å), Pi-alkyl: Tyr374 (4.25 Å), Tyr370 (5.12 Å), Trp321 (5.05 Å)	−5.6
*β*-Pinene	Alkyl: Val228 (5.00 Å), Leu265 (4.74 Å), Pro280 (5.04 Å)	−5.5	*β*-Pinene	Pi-sigma: Tyr71 (3.81 Å), Tyr370 (3.77 Å), Pi-alkyl: Tyr374 (5.21 Å), Trp83 (5.38 Å)	−7.1
Phytol	Alkyl: Met335 (3.97 Å), H-bond: Pro389 (2.47 Å). C-H bond: Thr390 (3.74 Å), Pi-alkyl: Phe381 (3.85 Å), Phe392 (4.91 Å)	−5.8	Phytol	H bond: Tyr324 (2.73 Å), Pi-alkyl: Tyr71 (4.88 Å), Trp321 (4.79 Å)	−5.9
Germacrene D	Alkyl: Val269 (4.67 Å)	−7.4	Germacrene D	Pi-sigma: Tyr71(3.78 Å)	−8.5
*γ*-Sitosterol	Alkyl: Pro280 (5.41 Å), Val228 (4.97 Å), Ile380 (5.04 Å), Met335 (5.07 Å), Leu427 (5.15 Å), Leu368 (4.37 Å), Pi-alkyl: Phe424 (5.38 Å), Phe381 (4.86 Å), Pi-sigma: Phe381(3.59 Å)	−10.4	*γ*-Sitosterol	H-bond: Leu62 (2.69 Å), Alkyl: Ala50 (4.79 Å), Pro33 (4.43 Å), Trp53 (5.46 Å) Pi-alkyl: Tyr95 (5.28 Å), Leu57 (4.97 Å)	−9.0
8 (17),12-Labdadiene-15,16-dial	Alkyl: Leu427 (5.46 Å), Pi-sigma: Phe381 (3.69 Å), Phe424 (3.89 Å), C-H bond: Glu394 (3.70 Å), H bond: Gln307 (2.22 Å)	−7.8	8 (17),12-Labdadiene-15,16-dial	H-bond: Tyr71 (2.86 Å)	−8.3
NTBC ^*∗*^	Pi-alkyl: Phe381 (5.02 Å) Pi-Pi stacked: Phe424 (4.78 Å), C-H bond: His308 (3.52 Å), H bond: Asn282 (2.22 Å), His308 (3.06 Å),	−8.9	Physostigmine ^*∗∗*^	H-bond: Tyr73 (2.73 Å), C-H bond: Asp375 (3.57 Å), Pi-donor hydrogen bond: Arg70 (3.39 Å), Pi-sigma: Trp321 (3.95 Å), Pi-Pi stacked: Trp321 (4.46 Å)	−7.4

NTBC: 2-[2-nitro-4-(trifluoromethyl) benzoyl] cyclohexane-1,3-dione,  ^*∗*^: known inhibitor of HPPD protein,  ^*∗∗*^ = known inhibitor of acetylcholinesterase.

## Data Availability

The data used to support the findings of this study are available from the corresponding authors upon request.
